# Multi-Oxidant Environment as a Suicidal Inhibitor of Myeloperoxidase

**DOI:** 10.3390/antiox12111936

**Published:** 2023-10-30

**Authors:** Ramona Clemen, Lara Minkus, Debora Singer, Paul Schulan, Thomas von Woedtke, Kristian Wende, Sander Bekeschus

**Affiliations:** 1 ZIK plasmatis, Leibniz Institute for Plasma Science and Technology (INP), Felix-Hausdorff-Str. 2, 17489 Greifswald, Germany; 2 Clinic and Policlinic for Dermatology and Venerology, Rostock University Medical Center, Strempelstr. 13, 18057 Rostock, Germany; 3 Institute for Hygiene and Environmental Medicine, Greifswald University Medical Center, Sauerbruchstr., 17475 Greifswald, Germany

**Keywords:** cold physical plasma, enzymatic activity, hydroxyl radical, kINPen, mass spectrometry, MPO, reactive oxygen species

## Abstract

Tissue inflammation drives the infiltration of innate immune cells that generate reactive species to kill bacteria and recruit adaptive immune cells. Neutrophil activation fosters the release of myeloperoxidase (MPO) enzyme, a heme-containing protein generating hypochlorous acid (HOCl) from hydrogen peroxide (H_2_O_2_) and chloride ions. MPO-dependent oxidant formation initiates bioactive oxidation and chlorination products and induces oxidative post-translational modifications (oxPTMs) on proteins and lipid oxidation. Besides HOCl and H_2_O_2_, further reactive species such as singlet oxygen and nitric oxide are generated in inflammation, leading to modified proteins, potentially resulting in their altered bioactivity. So far, knowledge about multiple free radical-induced modifications of MPO and its effects on HOCl generation is lacking. To mimic this multi-oxidant microenvironment, human MPO was exposed to several reactive species produced simultaneously via argon plasma operated at body temperature. Several molecular gas admixes were used to modify the reactive species type profiles generated. MPO was investigated by studying its oxPTMs, changes in protein structure, and enzymatic activity. MPO activity was significantly reduced after treatment with all five tested plasma gas conditions. Dynamic light scattering and CD-spectroscopy revealed altered MPO protein morphology indicative of oligomerization. Using mass spectrometry, various oxPTMs, such as +1O, +2O, and +3O, were determined on methionine and cysteine (Cys), and -1H-1N+1O was detected in asparagine (Asp). The modification types identified differed between argon-oxygen and argon-nitrogen plasmas. However, all plasma gas conditions led to the deamidation of Asp and oxidation of Cys residues, suggesting an inactivation of MPO due to oxPTM-mediated conformational changes.

## 1. Introduction

Neutrophils secrete myeloid peroxidase (MPO) during the oxidative burst, where aggressively lytic oxygen-centered radicals are secreted by these innate immune cells, supporting the first line of unspecific antimicrobial immune defense. MPO is known to generate hypobromous acid (HOBr), hypochlorous acid (HOCl), and hypothiocyanous acid (HOSCN) [1,2,3,4], which act as a disinfecting agent killing bacteria and pathogens. At the same time, MPO products also lead to lipid peroxidation [5] and can initiate oxidative tissue damage and cellular malfunction. For instance, circulating MPO initiates vascular inflammation and endothelium dysfunction and contributes to the progression of atherosclerosis. Therefore, intraplaque MPO activity is used as a biomarker to identify high-risk plaque [6], qualifying MPO inhibition as an interesting goal in drug research. Indeed, MPO inhibition reverts biomarker profiles strongly associated with heart failure [7]. Increasing evidence suggests that MPO drives the development of numerous other diseases correlating with chronic inflammation, such as Chron’s disease and ulcerative colitis [8], Alzheimer’s disease [9], cancer, and neurodegenerative disease [10]. Interestingly, in coronary artery disease patients, increased MPO concentrations were found in the blood, which correlated with elevated levels of circulating antibodies targeting oxidative post-translational protein modifications (oxPTMs) (e.g., protein-bound 3-nitrotyrosine) [11]. However, little is known about whether MPO gets modified by its own catalyzed reactive species and radicals, such as HOCl.

The 150 kDa enzyme MPO forms a dimer from two light chains (15 kDa) each and two heavy chains with different sizes depending on the isomer (57, 59, or 60 kDa). Different oxidized states of MPO are known for generating reactive species and are described in detail elsewhere [12]. In brief, in the resting state, the heme iron of MPO is in the ferric state, while oxidation generates a porphyrin p-cation radical species with an oxygen coupled to the heme iron in the ferryl state (compound I) via a double bond. Primary products are then produced via reduction to the native enzyme by abstracting two electrons from (pseudo-)halide ions (halogenation cycle), leading to HOCl, HOBr, HOSCN, or HOCN. Alternatively, two consecutive one-electron steps (peroxidase cycle) can lead to the generation of nitric oxide (NO) and nitrite (NO_2_^−^), tyrosyl radicals, and polyphenols. This formation of compound II contains a ferryl iron-oxygen moiety, and superoxide anion radicals can convert ferric MPO into compound III, a mixed ferric-superoxide/ferrous-dioxygen complex. Interestingly, a negative feedback loop of H_2_O_2_ on MPO was shown in vitro [13], and similar findings were observed in the presence of nitroxide [14,15]. During an immune response, a mixture of reactive species, including H_2_O_2_, is present [16,17], supporting the hypothesis of inhibiting and controlling MPO for homeostasis. However, no current data exist on whether a mixture of reactive oxygen and nitrogen species affects the protein’s activity.

Cold physical plasma (also called medical gas plasma, cold atmospheric pressure plasma, low-temperature plasma, non-thermal plasma, cold plasma; CAP, LTP, or NTP) simultaneously generates short-lived reactive oxygen and nitrogen species [18,19]. By coupling energy (e.g., electric fields) into a gas (e.g., argon, helium) ignition takes place, and the ionization of the gas atoms or molecules generates free electrons, gas ions, higher energy state neutrals, and radicals. The presence of oxygen, nitrogen, and water drives the formation of nitric oxide (NO), nitrite (NO_2_^−^), nitrate (NO_3_^−^) [20,21] or hydroxyl radicals (OH), H, and ^.^O [22]. Plasmas are standard in industrial processes and—more recently—emerged for biomedical applications. The plasma jet kINPen used in this study is an approved clinical device [23] with proven antimicrobial effects and wound closure promotion in chronic non-healing wounds [24,25]. Recently, we found altered immunogenicity of kINPen plasma-treated proteins [26,27] and inhibition of phospholipase A_2_ [28]. However, evidence is absent on whether plasma affects MPO. Therefore, the present study investigates the activity and structure of plasma-treated MPO, along with introducing oxidative post-translational modifications (oxPTMs) using high-resolution mass spectrometry. We found MPO changes depending on the reactive species mixture generated by physical plasma.

## 2. Materials and Methods

### 2.1. Protein Reconstitution

MPO (≥95% purity, >200 U per mg protein, negatively tested for HBsAg; HIV; HCV antibodies, Sigma Aldrich, Taufkirchen, Germany) was reconstituted following the manufacturer’s recommendations in dH_2_O (concentration of 1 mg mL^−1^). The MPO supplier’s datasheet can be found as Supplemental file. Aliquots (10 µL and 20 µL) of these solutions were stored at −80 °C until use. Before treatment, aliquots were thawed and diluted in phosphate-buffered saline (PBS), 50 mM potassium phosphate buffer, or 50 mM ammonium bicarbonate puffer (AmBiC). An amount of 100 µL protein suspension was added per well in a 96-well plate with properties optimal for suspension cells (Sarstedt, Sarstedt, Germany) for plasma treatment. 4-Aminobenzoic acid hydrazide (ABAH; Sigma Aldrich, Taufkirchen, Germany) served as positive control, and MPO was treated with 100 µM ABAH. Denaturation was carried out for 5 min at 70 °C.

### 2.2. Plasma Device and Treatment

The kINPen (neoplas, Greifswald, Germany) is an atmospheric pressure plasma jet operating at radiofrequency at a sinusoidal voltage waveform, ranging from 2 to 3 kV amplitude peak at a frequency of 1 MHz. In the jet, a ceramic capillary with an inner diameter of 1.6 mm has mounted a pin-type electrode at its center with a diameter of 1.0 mm. The cold plasma is generated at the tip of the central electrode, and the effluent expands into the ambient air. The kINPen was operated at flow rates of 1.0 to 1.5 standard liters per minute (slm) with argon or helium gas with admixtures of oxygen (0.5% for argon, 2% for helium) and/or nitrogen (0.5%). All gases were 99.999% pure (Air Liquide, Bremen, Germany). A homemade gas shielding device made of glass [29] (flushed with 3 slm N_2_) was attached around the jet for the 0.5% oxygen admixture setting, causing a near-laminar curtain gas flow around the jet effluent, suppressing its interaction with the ambient atmosphere. During all treatment conditions, the plasma effluent was in direct contact with the target surface (conducting mode) [30] or at the closest possible distance (when using the shielding device). The evaporated volume was filled up with dH_2_O (or HPLC water), and protein concentration was determined to ensure constant protein concentrations. Scavengers were freshly prepared in potassium phosphate buffer, added to the MPO solutions before the plasma treatment, and incubated for at least 10 min at room temperature. The final concentration of the scavenger was 2 mM 2-(4-Carboxyphenyl)-4,4,5,5-tetramethylimidazoline-1-oxyl-3-oxide (cPTIO; Dojindo Laboratories, Tokyo, Japan), 20 mM L-ergothioneine (Enzo Life Sciences, Lörrach, Germany), 20 mM histidine (Sigma-Aldrich, Taufkirchen, Germany), and 100 mM mannitol (Sigma-Aldrich, Taufkirchen, Germany).

### 2.3. MPO Activity Assays

**Figure 1:**
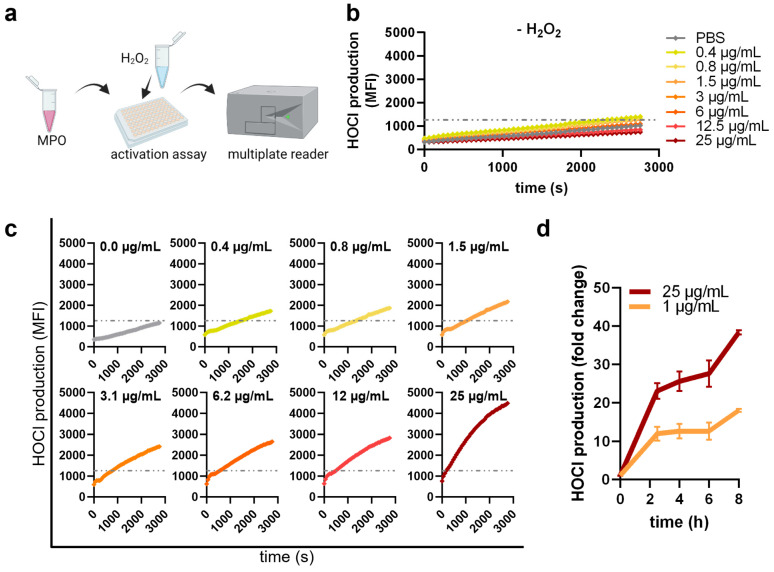
**Myeloperoxidase activity assay.** (**a**) Workflow of APF assay to determine hypochlorous acid based on the resulting reaction product of enzymatically active MPO diluted in PBS at indicated concentrations (**b**) without or (**c**) with adding 1 mM H_2_O_2_ and measuring for 45 min and (**d**) up to 8 h (for 1 µg/mL and 25 µg/mL MPO). Data are presented as the average of three experiments ± SEM.

Enzymatic activity was measured based on the formation of HOCl by the enzymatically active MPO (or oxidized MPO, respectively) from H_2_O_2_ and chloride ions (Figure 1a), assayed by 3,3′,5,5′-Tetramethylbenzidine (TMB; Carl Roth, Karlsruhe, Germany) or 3′-(p-aminophenyl) fluorescein (APF; Enzo Life Sciences, Lörrach, Germany), as indicated in the figure legend and text. For the TMB assay, 5 µL of the sample (10 µg/mL MPO) was combined with 80 µL H_2_O_2_ (0.75 mM) and 110 µL TMB solution (2.67 mM TMB in 14.5% DMSO and 150 mM sodium phosphate buffer with a pH value of 5.4). The solution was incubated in a 96-well plate for 3 min at 37 °C, and then the reaction was stopped by adding 50 μL of 2 M sulfuric acid. To assess the impact of plasma treatment on the enzyme activity, MPO solutions were plasma-treated at least in duplicate, and each of the duplicates was then measured in triplicate. The APF assay was performed in a 96-well plate with 90 µL PBS containing 5 µg/mL MPO per well. Then, 5 µL of 100 µM APF solution and 5 µL H_2_O_2_ were added. A kinetic experiment was started measuring the fluorescence at an λ_ex_ 485 nm and λ_em_ 535 nm using a microplate reader (Infinite F200 pro; Tecan, Männedorf, Switzerland).

### 2.4. High-Resolution LC-MS2 Measurements of MPO

MPO was diluted in ammonium bicarbonate buffer (10 µg/mL) and exposed to plasma (Ar, Ar/O_2_, Ar/N_2_) for 30 s. Subsequently, the protein was digested by trypsin V5111 (Promega, Walldorf, Germany) (ratio: MPO/trypsin, 40:1) and subjected to nano-liquid chromatography mass spectrometry (nLC-MS). Peptides (100 ng) were loaded onto a PepMap C18 trap column. A Dionex UltiMate 3000 RSLCnano HPLC was connected to an Exploris 480 mass spectrometer using a Nanospray Flex ion source (ThermoFisher, Dreieich, Germany). Peptides were eluted from the trap column and separated on a 150 mm × 75 µm ID Acclaim PepMap C18 column using buffer A (0.1% *v*/*v* acetic acid) and buffer B (95:5 acetonitrile: 0.1% *v*/*v* acetic acid) at a flow rate of 300 nL/min with an elution gradient of 4–40% buffer B over 50 min. The column temperature was set to 40 °C. The mass spectrometer was run in positive polarity mode with a transfer capillary temperature of 250 °C and a spray voltage of 2 kV. Spectra were recorded in DDA acquisition mode (Top 15), and peptides were analyzed in full scan (350–1200 m/z, R = 120,000 at 200 m/z) with a target of 5 × 10^3^ ions, followed by 15 data-dependent MS/MS scans with higher energy collisional dissociation (HCD, maximum injection time (IT) 50 ms, isolation width 1.0 m/z, NCE 30%), detected in orbitrap (R = 15,000 at 200 m/z). Dynamic exclusion was enabled and set to 30 s.

### 2.5. LC-MS/MS Data Analysis

Raw LC-MS/MS data were analyzed with Proteome Discoverer software, version 2.4.1.15 (ThermoFisher, Dreieich, Germany). MS/MS spectra were extracted from the raw files, and search was performed using custom FASTA files, each containing a single protein sequence. MPO was unambiguously identified (79% coverage, 90 Peptides, 13272 PSMs, and 2265 isoforms total found). PMI-Byonic (Protein Metrics, Cupertino, CA, USA) was used for the identification and quantification of PSM and PTM. The search engine parameters for PMI-Byonic were set as follows: peptide mass tolerance = 10 ppm, fragment mass tolerance = 10 ppm, cleavage specificity = trypsin, missed cleavages = 2, and total common modifications = 2. A custom modification list was used based on previous experiments [30] (Figure S2). The raw data were filtered using Perseus 2.0.3.0 software and modifications were considered valid if a protein modification occurred in ≥66% replicates of a group. The valid dataset was imported into Excel, and the ratio of modified peptide sites to unmodified and otherwise modified peptide sites in the entire dataset was calculated for each modification. The cut-off for the ratio was set at ≥0.1; i.e., at least 10% of this peptide site was modified. The significance of the modification was determined using a custom R script (2022.02.1-461) that performed a Wilcoxon rank sum test with a cut-off of *p* ≤ 0.05.

### 2.6. Photon Correlation Spectroscopy

Particle size analysis of MPO and plasma-treated MPO was carried out using a ZS90 dynamic light scattering (DLS) device (Malvern Panalytical, Kassel, Germany) equipped with a helium-neon laser light source (632 nm). Proteins (material RI = 1.45, absorption = 0.001) in a buffer (dispersant RI = 1.33, viscosity = 0.954) were measured in low-volume cuvettes (70 µL). All identical samples and controls were concentrated to twice the concentration (200 µg/mL) using a vacuum concentrator, pooled, and centrifuged at 21,000× *g* and 4 °C for 2.5 h. DLS measurements were carried out at a set angle of 90° and attenuator at 11. Size was measured at 22 °C with an equilibration time of 120 s and a cuvette position at 3 mm. Backscatter-angled detection was performed at 173° with a scattering collection angle of 147.7°. The measurement was repeated three times, and each replicate was measured in several replicates with minimal time between repeats.

### 2.7. Circular Dichroism (CD) Spectroscopy

MPO and oxidized MPO (1 mg/mL) measurements were performed in 1 mm cuvettes using a Chirascan CD spectrometer (Applied Photophysics, Leatherhead, England) coupled to a temperature controller. The CD spectra were recorded at 25 °C from 190 to 250 nm. The bandwidth was 1 nm, and the scan time per point was 1.5 s. CD analysis was measured in four replicates, each containing two pooled technical replicates. All spectra were blank-corrected.

### 2.8. SDS Page and Silver Staining

Denaturated (5 min, 95 °C) proteins (35 µg) were loaded with 4× sample buffer on a 10% polyacrylamide gel. Then, 10 µL of a prestained protein ladder with a 10 to 250 kDa size range was used as a protein standard (all Thermo Fisher, Dreieich, Germany). The loaded gels were run in a gel electrophoresis chamber with a running buffer, first at 50 V for 20 min and then at 120 V for 60 min. MPO and plasma-treated MPO were visualized using the ready-to-use Silver Stain Plus Kit according to the manufacturer’s instructions (Bio-Rad Laboratories, Düsseldorf, Germany).

### 2.9. Statistical Analysis

Graphing and statistical analysis were performed using GraphPad Prism 9.5.1 (GraphPad Software, San Diego, CA, USA). Statistical comparisons and the tests employed are referenced in the figure legend. Levels of significance are indicated as follows: *p* < 0.05 (*), *p* < 0.01 (**), *p* < 0.001 (***).

## 3. Results

### 3.1. Plasma Treatment Decreased MPO Activity

First, the enzymatic activity of MPO was determined in the presence of APF reagent with or without adding H_2_O_2_ (Figure 1a). Different MPO concentrations were tested to determine correlations with the MPO amount and its HOCl generation within 45 min. No MPO activity without adding H_2_O_2_ was observed (grey line) (Figure 1b). When adding H_2_O_2_, MPO started to generate HOCl and enzyme concentrations lower than 3 µg/mL needed 1000 s to reach signals higher than PBS control (Figure 1c). Higher MPO concentrations started to generate HOCl immediately after adding 1 mM H_2_O_2_ and the maximum signal for 25 µg/mL MPO was 2.6 times higher compared to 0.4 µg/mL MPO. We further measured the HOCl production with 1 µg/mL and 25 µg/mL MPO after prolonged incubation times of up to 8 h and observed increasing amounts of HOCl (Figure 1d). However, an unbalanced system using other ratios of APF and H_2_O_2_ led to saturation and a low concentration of APF (1 µM) showed best signal-to-noise ratios after 4 h (Figure S1a,b).

**Figure 2:**
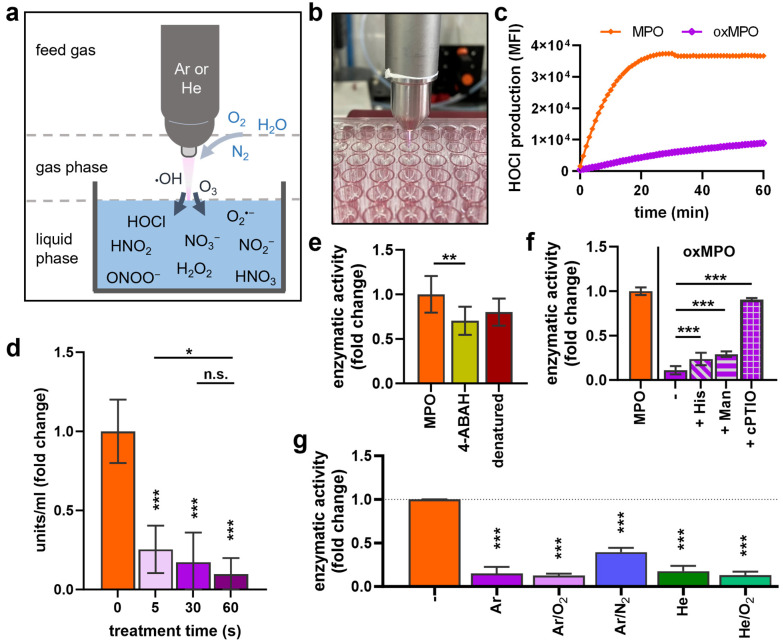
**MPO activity decreased after plasma treatment.** (**a**) Scheme of physical plasma technology ionizing feed gas and releasing reactive species in the gas phase that further react with a treated surface (here: liquid). (**b**) Image of argon plasma treatment of MPO (oxMPO) in PBS before (**c**) APF and H_2_O_2_ were added to measure HOCl production for 60 min due to MPO activation. (**d**) MPO exposure to different argon plasma treatment times and TMB-based MPO activity calculation. (**e**) MPO was treated with 4-Aminobenzoic acid hydrazide (ABAH, 100 µM final concentration) or incubated for 5 min at 70 °C (denaturation) before H_2_O_2_ was added, and HOCl was measured by APF assay. (**f**) Histidine (His, 20 mM), mannitol (Man, 100 mM), or cPTIO (2 mM) were added prior to plasma treatment, and MPO activity was measured via TMB assay. (**g**) MPO was exposed to different plasma gas conditions for 30 s, and enzymatic activity measurement via TMB and APF assays. Data (**d**–**g**) are mean ± SEM normalized to untreated control from two to three independent experiments with three to six replicates each. Statistical analysis was performed using Mann–Whitney test (n.s. = not significant, * *p* < 0.05, ** *p* < 0.01, *** *p* < 0.001).

Plasma-generated reactive gas phase species are either transported into the liquid or react with molecules and ions present, forming secondary species (Figure 2a). The extent increases when the plasma effluent touches the liquid (conductive mode), as was the case in the current approach [30]. MPO was exposed to plasma in a 96-well plate for different durations (Figure 2b). Kinetic APF measurements revealed reduced MPO activity when the enzyme was plasma-treated for 20 s (oxMPO) before 100 µM H_2_O_2_ was added (Figure 2c). This reduction was observed continuously for 60 min. Importantly, lower H_2_O_2_ concentration (10 µM) resulted in reduced MPO activity within the first 50 min but reached similar HOCl signals to untreated MPO after 60 min (Figure S1d). Plasma-induced MPO activity reduction was confirmed with TMB assay, where enzymatic activity after different exposure times was tested. A stronger reduction occurred after prolonged treatment times (Figure 2d). While plasma lowered the activity to a residual ≤ 20%, treatment with the inhibitor 4-aminobenzoic acid hydrazide (ABAH) or protein denaturation reduced the MPO activity only to 75% (Figure 2e). To investigate which of the plasma-generated reactive species was responsible for the reduced enzymatic activity, scavengers were used. The ^1^O_2_ scavenger L-Histidine and ^.^OH scavenger mannitol rescued MPO activity reduction modestly but significantly (Figure 2f). However, the NO scavenger cPTIO fully abrogated plasma-induced MPO activity reduction. A reduction in the enzyme activity was also observed with other plasma feed gas mixtures, finding that Ar/N_2_ plasmas decreased the activity to the least extent (Figure 2g).

### 3.2. Plasma Treatment Modified the Protein Structure of MPO

**Figure 3:**
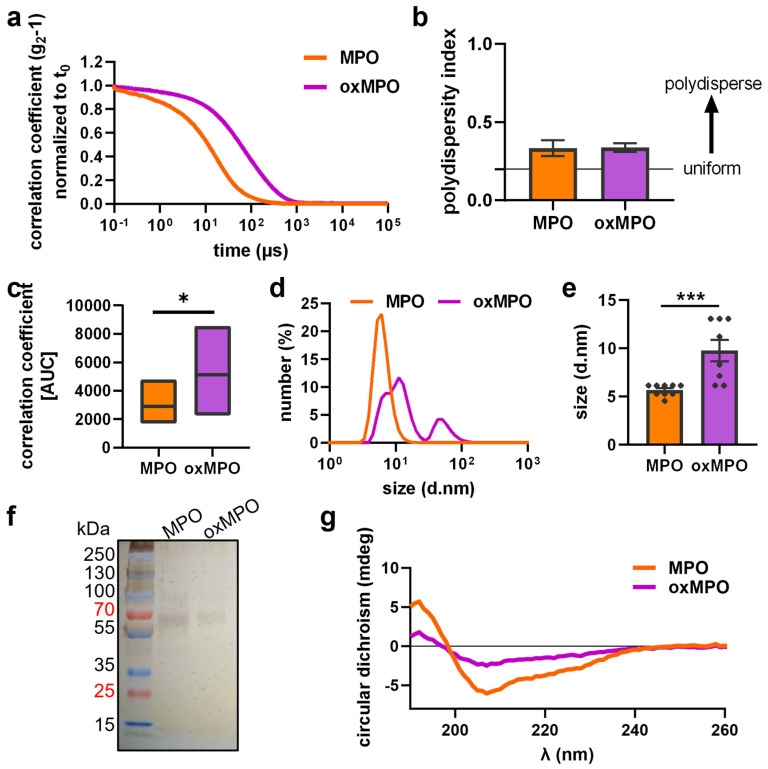
**MPO structural changes were identified after plasma treatment.** MPO was treated with argon plasma, and structural changes were measured. Dynamic light scattering revealed changes in (**a**,**c**) correlation coefficient, (**b**) polydispersity index, and (**d**) measured and (**e**) quantified size. SDS page (**f**), followed by silver staining, did not show oligomerization, and (**g**) shift in secondary structure was determined via circular dichroism spectroscopy. Data are shown as the mean of three experiments ± SEM. Statistical analysis was performed using *T*-test (* *p* < 0.05, *** *p* < 0.01).

We next investigated structural changes to MPO after exposure to argon plasma in PBS by photon correlation spectroscopy, CD spectroscopy, and gel electrophoresis. Plasma treatment drove MPO into aggregation/oligomerization, as indicated by an altered correlation coefficient (Figure 3a,c). DLS measurement revealed that a uniform population of aggregates appeared (Figure 3b). A more than two-fold size increase was observed (Figure 3d,e). Assuming that plasma treatment may trigger dimerization, we performed SDS PAGE. However, only monomers were observed for native and oxidized MPO. No size shift for stably formed dimers or oligomers were visible (Figure 3f). We further examined the MPO protein structure via CD spectroscopy, measuring α-helical structures indicated by a minimum signal at 209 nm and a shoulder at 222 nm; and β-sheets showing peaks at 195 nm and a negative minimum at 217 nm. We identified structural changes after plasma treatment and shrinking signals of the proportionate α- and β-structures in MPO (Figure 3g). To investigate if structural changes occur due to changes in specific amino acids that are relevant for protein folding, we next performed mass spectrometry to identify oxPTMs.

### 3.3. Plasma Treatment Provoked oxPTMs

**Figure 4:**
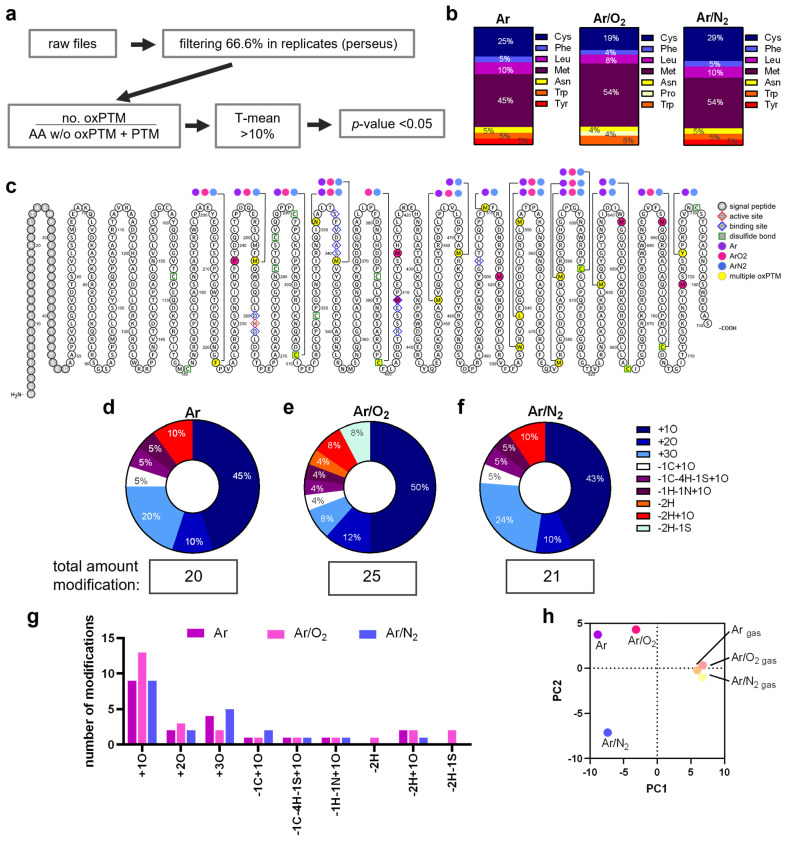
**MPO oxPTMs were detected after plasma treatment.** MPO was exposed to Ar, Ar/O_2_, or Ar/N_2_ plasmas, or gas controls (plasma = off). Samples of three experiments were trypsin-digested and subjected to solid-phase extraction as described in the Materials and Methods section before LC-MS/MS analysis. (**a**) Data evaluation workflow: sorted raw files were analyzed via proteome discoverer and filtered using Perseus software and R script to identify ≥10% valid oxPTMs occurring in ≥66.6% of replicates. (**b**) oxPTM distribution per amino acid. (**c**) Human MPO amino acid sequence and oxPTMs determined after treatment with Ar (purple), Ar/O_2_ (pink), and Ar/N_2_ (blue) plasma. (**d**–**f**) MPO oxPTM distribution (shown without digits after decimal) after exposure to (**d**) Ar, (**e**) Ar/O_2_, and (**f**) Ar/N_2_ plasma. (**g**) Corresponding number of individual oxPTMs. (**h**) Principal component analysis of modification data, showing similarities in control gas-treated samples, while plasma-treated MPO samples differed.

As previous studies revealed, protein-bound amino acids show dramatically different rate constants for chemical reactions with reactive species, yielding to a variety of oxPTMs types and their abundances [31,32]. MPO contains 745 amino acids, including 17 cysteines, 19 tyrosines, and 23 methionine, which represent preferred targets for oxidation. After plasma exposure and subsequent protease digestion, LC-MS/MS was performed to map the newly introduced oxPTMs (Figure 4a and Figure S2). The amino acids Cys, Met, Tyr, and Trp have been shown to exhibit different susceptibility to modifications by Ar and Ar/O_2_ plasmas [33], which is also reflected in the current data (Figure 4b). Figure 4c gives an overview on the observed oxPTMs in the amino acid sequence of MPO. Individual positions were marked in color for oxPTM that occur after treatment with Ar (purple), Ar/O_2_ (pink), or Ar/N_2_ (blue) plasmas. OxPTMs that occurred in multiple plasma conditions were highlighted in yellow and labeled separately.

The amino acids Cys, Phe, Leu, Met, and Asn showed a fairly similar susceptibility towards different plasma conditions and the respective mix of reactive species generated, except for proline, which was modified only after exposure to Ar/O_2_ plasma. On the contrary, Ar/O_2_ plasma did not result in any observed Tyr modifications, albeit Tyr was modified by the exposure to Ar and Ar/N_2_ plasmas (Figure 4b). Interestingly, Cys 309, Cys 398, Cys 606, Cys 663, and Cys 704 that are all involved in disulfide binding showed oxPTMs. Looking in more detail at the specific oxPTMs that were induced (Figure S3), we found that 43–50% of all modifications were oxidation (+1O) and 10–12% dioxidation (+2O) (Figure 4d–f). Further modifications (-1C+1O; -C-4H-1S-1O; -1H-1N+1O; -2H+1O) were also found in similar proportions after plasma treatment with all conditions, but dehydration (-2H, orange) and the loss of the thiol group (-H2S, green) were found only after treatment with Ar/O_2_ plasma. Interestingly, the proportion of trioxidation was similar after treatment with Ar/N_2_ (24%) and Ar (20%) plasmas but not after Ar/O_2_ plasma exposure (8%). Those proportions were also reflected in the total number of modifications (Figure 4g). A principal component analysis of the observed oxPTM compositions revealed a good agreement of the gas controls (Ar, Ar/O_2_, or Ar/N_2_; plasma = off, oxidation background) that clustered together, while oxidized MPO treated with the different plasma conditions differed strongly from each other and the controls (Figure 4h).

## 4. Discussion

MPO is relevant for pathogen clearance [34] by generating reactive oxygen species. On the other hand, MPO correlates with several diseases, making it a suitable target for inhibition. Indeed, the irreversible MPO inhibitor AZD3241 improved the clinical outcome in a mice study for chronic inflammatory bowel disease [35] but failed for treatment in multiple system atrophy [36]. Cold plasma technology probably does not meet the requirements of a drug for treating MPO-related disease because systemic MPO inhibition is not possible with a local treatment. However, the technology allows the mimicking of an inflammatory response and the investigation of reactive species impact on the structure and functionality of engaged proteins.

Reactive species can activate or inhibit proteins due to the induction of oxPTMs, such as in the case of the thiol-based redox switches [37]. Along these lines, reactive species derived from cold physical plasma were shown to induce oxPTMs on peptides and proteins [38], leading to an altered activity [39,40]. The most prominent reactive species at the side of a physiologic inflammatory response is hydrogen peroxide. Interestingly, H_2_O_2_ has been found to inhibit MPO at a concentration of 1.5 mM [13], and our results align with these results when using lower APF concentrations (Figure S1a). However, it is unlikely that H_2_O_2_ is the only species relevant for MPO’s inactivation since He and HeO_2_ plasma also inactivate MPO, but these plasma conditions do not produce H_2_O_2_ [27]. H_2_O_2_ can decompose to hydroxyl radicals via the Fenton reaction and ^.^OH is generated in most plasma conditions via the cleavage of trace amounts of water. However, the OH scavenger mannitol was not able to rescue the inhibitory effect on MPO.

On the other hand, we find rescuing effects of NO scavenger cPTIO on argon plasma-treated MPO pointing to NO as one major contributor for protein oxidation by plasma. However, NO itself is not very reactive, and few oxPTMs can be attributed to its activity. Among these is the nitrosylation of free thiol groups that has been observed in a minute extent after plasma treatment of cysteine [41]. Accordingly, other reactive species that derive from NO come into focus. Among these, peroxynitrite ONOO^−^ is of note [42]. Having a relatively long lifetime combined with a high reactivity in acidic conditions, it may be responsible for many of the oxPTMs observed in MPO. The formation of ONOO^−^ as a tertiary species can be achieved via gas–liquid interface reactions between nitric oxide and singlet oxygen (^1^O_2_) or in the liquid bulk via the reaction of H_2_O_2_ with NO. Since this reaction is driven only in strongly acidic conditions (pH 3.3) [43], it can be assumed negligible. Ultimately, a reaction between nitric oxide and superoxide O_2_^−^ can also yield peroxynitrite (^.^NO + O_2_^−^ -> ONOO^−^) [44]. Superoxide is created in the gas phase of most discharges from electron attachment to molecular oxygen, and it has frequently been observed in plasma-treated liquids [45]. This is in line with the investigated argon plasma conditions, which reduced the enzymatic activity but induced different oxPTM on MPO, suggesting a general inhibitory effect of excessive oxidation on MPO activity.

In mature MPO, the amino acids Asp94, His95, Glu242, Met243, and His336 are major features of the MPO heme-binding pocket [46], and H_2_O_2_ triggers autocatalytic reactions [47]. Due to including signal peptide and propeptide sequence, these amino acids correspond to our MPO’s sequence Asp260, His261, Glu409, Met410, and His502. However, we did not identify any changes in these amino acids, with the exception of Met410, which showed an additional modification (-2H+2O) after one of the plasma conditions and dioxidation (+2O) after treatment with Ar/O_2_. Therefore, we conclude that plasma oxidation does not influence hem binding and probably does not promote autocatalytic reactions. We mainly identified oxidation on Met with all tested conditions, with slight variations. While Ar/O_2_ plasma modified 11 Met residues (at positions 341, 409, 415, 453, 519, 551, 577, 588, 644, 688, 719), the other plasmas modified only 8 Met. We further determined dioxidized and trioxidized cysteines, indicating the cleavage of disulfide bonds. Disulfide bridges are critical stabilizing motifs in maintaining the secondary structure of proteins, and previous studies have reported structural changes due to oxidative damage [48,49] and plasma treatment [39]. Importantly, the observed conformational changes are not similar to denatured MPO since the inhibitory effect after denaturation was less pronounced than the inhibitory effects of plasma. During the maturation process of MPO, disulfide binding in proMPO is a crucial step [50], so breaking disulfide bounds may drive MPO into a proMPO-like conformation. Furthermore, labile disulfides may enable the formation of hydrogen bonds due to reactive residues on cysteine and methionine due to altered electrostatic properties [51]. The changes in the protein’s architecture detected via CD spectroscopy after plasma treatment are consistent with this hypothesis.

Moreover, DLS measurement revealed a substantial increase in the size after plasma treatment, suggesting MPO homo-multimerization that can be reversed under denaturing and reducing conditions during SDS page. However, no differences in MPO’s chlorinating activity have yet been found in monomeric and dimeric forms of MPO [52], suggesting greater relevance to the combinatorial effect of amino acid modification, structural changes, and multimer formation. Myeloperoxidase’s di-Tyrosine (di-Tyr) formation occurs in the presence of chloride ions and L-tyrosine, leading to the cross-linking of proteins [53], similarly to other proteins as horseradish peroxidase (HRP) [54]. Furthermore, MPO was shown to multimerize α-Synuclein in the presence of H_2_O_2_ and NO_2_^−^ [55]. However, studies about MPO homodimerization are lacking, but it could be an interesting aspect of the pathogenesis of some diseases. For instance, the cross-linking dimer species was also found for amyloid-beta [56] and is suggested in amyloid plaques in Alzheimer’s disease brain section [57]. Hence, MPO could oxidize and form a homodimer or cross-link to other proteins, causing it to be inactivated and circulating in the bloodstream as a non-functional protein, bypassing the immune cells’ self-tolerance and accumulating eventually. In atherosclerotic lesions of animals, Zeng et al. found increased MPO expression correlating with di-Tyr, nitrotyrosine (NO_2_Tyr) and 3-chlorotyrosine [58], and Baldus and colleagues also found colocalization of NO_2_Tyr and MPO [59]. Other studies provide evidence of tyrosine nitration in proteins in human atherosclerotic lesions [60]. However, these studies did not investigate whether MPO is oxidatively modified or if the generated reactive species modify other proteins. More evidence is needed to identify MPO as the cause for oxPTM on other proteins or a possible victim of its self-generated species.

The hypothesis of oxidative dimerization of phenols, including protein Tyr residues, would support the results of previous preclinical and clinical studies where co-localization of MPO and Tyr-modification was found. Interestingly, nitrotyrosine formation by plasma treatment was identified previously and prevented by cPTIO [55], supporting that cPTIO recovers MPO activity. However, we did not identify oxPTMs on Tyr after treatment with Ar/O_2_, albeit the enzymatic activity was disrupted. Since tyrosin modifications were found in all other conditions, it can be assumed that the Ar/O_2_ treatment led to unidentified modifications that were not captured by the software algorithm. Another reason for dimerization might be the deamidation on Asn 133, as occurs in MPO after exposure to all plasma conditions. Deamidation would suggest a hydrogen bonding shift by removing NH residue and adding O (−1H−1N+1O), leading to acidic residue formation and protein dimerization. Interestingly, conversions of Asn residues to aspartic acid through deamidation and aspartate isomerization (IsoAsp) have been reported to affect protein stability [61,62]. Aspartic acid is believed to serve as a molecular clock for biological processes, seriously disrupting biologically important functions. Indeed, IsoAsp was shown to have increased immunogenicity and promote an immune response [63,64].

Further aspects that would help to understand the inhibitory effects of multiple reactive species on MPO are the analysis of structural changes, multimerization, and identification of specific radicals. For instance, investigating protein melting points due to the modifications would give insights into the folding. However, we can rule out that plasma fully denatures the protein since (1) kINPen plasma operates at body temperature and (2) the heat-denatured protein did not show any comparable restrictions in activity. The identification of dimerization in combination with reactive species would clarify the inactivation through a Western blot, which is more sensitive than silver staining. However, a Western blot would still require that the samples run in SDS gel, and unstable dimerization can break down due to the reducing conditions. A compound for the controlled multimerizing of MPO has not yet been described in the literature, and a similar situation is true for individual radicals as positive controls for protein oxidation. Since our study aims at mimicking an inflammatory response and not the effect of a single reactive species, their use is limited anyway. During inflammation, a mixture of several species is present simultaneously, and physical plasma technology can generate various short-lived species. Altering the gas fed into the plasma jet used in this study even changes the reactive species composition produced with this plasma.

Altogether, our study provides evidence that NO/ONOO^−^ and OCl^−^ are more important for MPO inactivation compared to OH, and our data suggest that amino acid oxidation at multiple sites may be linked to conformational changes and reduced activity observed after MPO plasma treatment.

## Data Availability

The underlying data can be received from the corresponding author upon reasonable request.

## Supplementary Materials

The following supporting information can be downloaded at: https://www.mdpi.com/article/10.3390/antiox12111936/s1.

## References

[1] Winterbourn C.C., Kettle A.J., Hampton M.B. (2016). Reactive Oxygen Species and Neutrophil Function. Annu. Rev. Biochem..

[2] Davies M.J. (2011). Myeloperoxidase-derived oxidation: Mechanisms of biological damage and its prevention. J. Clin. Biochem. Nutr..

[3] Ulfig A., Leichert L.I. (2021). The effects of neutrophil-generated hypochlorous acid and other hypohalous acids on host and pathogens. Cell. Mol. Life Sci..

[4] Hampton M.B., Kettle A.J., Winterbourn C.C. (1998). Inside the neutrophil phagosome: Oxidants, myeloperoxidase, and bacterial killing. Blood.

[5] Zhang R., Brennan M.L., Shen Z., MacPherson J.C., Schmitt D., Molenda C.E., Hazen S.L. (2002). Myeloperoxidase functions as a major enzymatic catalyst for initiation of lipid peroxidation at sites of inflammation. J. Biol. Chem..

[6] Nadel J., Tumanov S., Kong S.M.Y., Chen W., Giannotti N., Sivasubramaniam V., Rashid I., Ugander M., Jabbour A., Stocker R. (2023). Intraplaque Myeloperoxidase Activity as Biomarker of Unstable Atheroma and Adverse Clinical Outcomes in Human Atherosclerosis. JACC Adv..

[7] Michaelsson E., Lund L.H., Hage C., Shah S.J., Voors A.A., Saraste A., Redfors B., Grove E.L., Barasa A., Richards A.M. (2023). Myeloperoxidase Inhibition Reverses Biomarker Profiles Associated with Clinical Outcomes in HFpEF. JACC Heart Fail..

[8] Swaminathan A., Borichevsky G.M., Edwards T.S., Hirschfeld E., Mules T.C., Frampton C.M.A., Day A.S., Hampton M.B., Kettle A.J., Gearry R.B. (2022). Faecal Myeloperoxidase as a Biomarker of Endoscopic Activity in Inflammatory Bowel Disease. J. Crohns Colitis.

[9] Smyth L.C.D., Murray H.C., Hill M., van Leeuwen E., Highet B., Magon N.J., Osanlouy M., Mathiesen S.N., Mockett B., Singh-Bains M.K. (2022). Neutrophil-vascular interactions drive myeloperoxidase accumulation in the brain in Alzheimer’s disease. Acta Neuropathol. Commun..

[10] Davies M.J., Hawkins C.L. (2020). The Role of Myeloperoxidase in Biomolecule Modification, Chronic Inflammation, and Disease. Antioxid. Redox Signal..

[11] Thomson L., Tenopoulou M., Lightfoot R., Tsika E., Parastatidis I., Martinez M., Greco T.M., Doulias P.T., Wu Y., Tang W.H. (2012). Immunoglobulins against tyrosine-nitrated epitopes in coronary artery disease. Circulation.

[12] Arnhold J., Flemmig J. (2010). Human myeloperoxidase in innate and acquired immunity. Arch. Biochem. Biophys..

[13] Paumann-Page M., Furtmuller P.G., Hofbauer S., Paton L.N., Obinger C., Kettle A.J. (2013). Inactivation of human myeloperoxidase by hydrogen peroxide. Arch. Biochem. Biophys..

[14] Kajer T.B., Fairfull-Smith K.E., Yamasaki T., Yamada K., Fu S., Bottle S.E., Hawkins C.L., Davies M.J. (2014). Inhibition of myeloperoxidase- and neutrophil-mediated oxidant production by tetraethyl and tetramethyl nitroxides. Free Radic. Biol. Med..

[15] Maiocchi S., Ku J., Hawtrey T., De Silvestro I., Malle E., Rees M., Thomas S.R., Morris J.C. (2021). Polyamine-Conjugated Nitroxides Are Efficacious Inhibitors of Oxidative Reactions Catalyzed by Endothelial-Localized Myeloperoxidase. Chem. Res. Toxicol..

[16] Steiling H., Munz B., Werner S., Brauchle M. (1999). Different types of ROS-scavenging enzymes are expressed during cutaneous wound repair. Exp. Cell Res..

[17] Niethammer P., Grabher C., Look A.T., Mitchison T.J. (2009). A tissue-scale gradient of hydrogen peroxide mediates rapid wound detection in zebrafish. Nature.

[18] Gorbanev Y., Privat-Maldonado A., Bogaerts A. (2018). Analysis of Short-Lived Reactive Species in Plasma-Air-Water Systems: The Dos and the Do Nots. Anal. Chem..

[19] Schmidt-Bleker A., Winter J., Iseni S., Dunnbier M., Weltmann K.D., Reuter S. (2014). Reactive species output of a plasma jet with a shielding gas device-combination of FTIR absorption spectroscopy and gas phase modelling. J. Phys. D Appl. Phys..

[20] Zhang K., Zhao M., Sun D.-W., Tiwari B.K. (2023). Correlation of plasma generated long-lived reactive species in aqueous and gas phases with different feeding gases. Plasma Sources Sci. Technol..

[21] Bekeschus S., Schmidt A., Niessner F., Gerling T., Weltmann K.D., Wende K. (2017). Basic Research in Plasma Medicine—A Throughput Approach from Liquids to Cells. J. Vis. Exp..

[22] Takamatsu T., Uehara K., Sasaki Y., Miyahara H., Matsumura Y., Iwasawa A., Ito N., Azuma T., Kohno M., Okino A. (2014). Investigation of reactive species using various gas plasmas. Rsc. Adv..

[23] Bekeschus S., von Woedtke T., Emmert S., Schmidt A. (2021). Medical gas plasma-stimulated wound healing: Evidence and mechanisms. Redox Biol..

[24] Isbary G., Morfill G., Schmidt H.U., Georgi M., Ramrath K., Heinlin J., Karrer S., Landthaler M., Shimizu T., Steffes B. (2010). A first prospective randomized controlled trial to decrease bacterial load using cold atmospheric argon plasma on chronic wounds in patients. Br. J. Dermatol..

[25] Isbary G., Heinlin J., Shimizu T., Zimmermann J.L., Morfill G., Schmidt H.U., Monetti R., Steffes B., Bunk W., Li Y. (2012). Successful and safe use of 2 min cold atmospheric argon plasma in chronic wounds: Results of a randomized controlled trial. Br. J. Dermatol..

[26] Clemen R., Arlt K., Miebach L., von Woedtke T., Bekeschus S. (2022). Oxidized Proteins Differentially Affect Maturation and Activation of Human Monocyte-Derived Cells. Cells.

[27] Clemen R., Freund E., Mrochen D., Miebach L., Schmidt A., Rauch B.H., Lackmann J.W., Martens U., Wende K., Lalk M. (2021). Gas Plasma Technology Augments Ovalbumin Immunogenicity and OT-II T Cell Activation Conferring Tumor Protection in Mice. Adv. Sci..

[28] Nasri Z., Memari S., Wenske S., Clemen R., Martens U., Delcea M., Bekeschus S., Weltmann K.D., von Woedtke T., Wende K. (2021). Singlet Oxygen-Induced Phospholipase A2 Inhibition: A Major Role for Interfacial Tryptophan Dioxidation. Chemistry.

[29] Reuter S., Winter J., Schmidt-Bleker A., Tresp H., Hammer M.U., Weltmann K.D. (2012). Controlling the Ambient Air Affected Reactive Species Composition in the Effluent of an Argon Plasma Jet. IEEE Trans. Plasma Sci..

[30] Miebach L., Freund E., Cecchini A.L., Bekeschus S. (2022). Conductive Gas Plasma Treatment Augments Tumor Toxicity of Ringer’s Lactate Solutions in a Model of Peritoneal Carcinomatosis. Antioxidants.

[31] Davies M.J. (2003). Singlet oxygen-mediated damage to proteins and its consequences. Biochem. Biophys. Res. Commun..

[32] Wilkinson F., Helman W.P., Ross A.B. (1995). Rate Constants for the Decay and Reactions of the Lowest Electronically Excited Singlet-State of Molecular-Oxygen in Solution—an Expanded and Revised Compilation. J. Phys. Chem. Ref. Data.

[33] Wenske S., Lackmann J.W., Busch L.M., Bekeschus S., von Woedtke T., Wende K. (2021). Reactive species driven oxidative modifications of peptides-Tracing physical plasma liquid chemistry. J. Appl. Phys..

[34] Aratani Y., Koyama H., Nyui S., Suzuki K., Kura F., Maeda N. (1999). Severe impairment in early host defense against *Candida albicans* in mice deficient in myeloperoxidase. Infect. Immun..

[35] Ahmad G., Chami B., Liu Y., Schroder A.L., San Gabriel P.T., Gao A., Fong G., Wang X., Witting P.K. (2020). The Synthetic Myeloperoxidase Inhibitor AZD3241 Ameliorates Dextran Sodium Sulfate Stimulated Experimental Colitis. Front. Pharmacol..

[36] Kaindlstorfer C., Sommer P., Georgievska B., Mather R.J., Kugler A.R., Poewe W., Wenning G.K., Stefanova N. (2015). Failure of Neuroprotection Despite Microglial Suppression by Delayed-Start Myeloperoxidase Inhibition in a Model of Advanced Multiple System Atrophy: Clinical Implications. Neurotox. Res..

[37] Groitl B., Jakob U. (2014). Thiol-based redox switches. Biochim. Biophys. Acta.

[38] Wenske S., Lackmann J.W., Bekeschus S., Weltmann K.D., von Woedtke T., Wende K. (2020). Nonenzymatic post-translational modifications in peptides by cold plasma-derived reactive oxygen and nitrogen species. Biointerphases.

[39] Lackmann J.W., Baldus S., Steinborn E., Edengeiser E., Kogelheide F., Langklotz S., Schneider S., Leichert L.I.O., Benedikt J., Awakowicz P. (2015). A dielectric barrier discharge terminally inactivates RNase A by oxidizing sulfur-containing amino acids and breaking structural disulfide bonds. J. Phys. D Appl. Phys..

[40] Krewing M., Stepanek J.J., Cremers C., Lackmann J.W., Schubert B., Muller A., Awakowicz P., Leichert L.I.O., Jakob U., Bandow J.E. (2019). The molecular chaperone Hsp33 is activated by atmospheric-pressure plasma protecting proteins from aggregation. J. R. Soc. Interface.

[41] Lackmann J.W., Bruno G., Jablonowski H., Kogelheide F., Offerhaus B., Held J., Schulz-von der Gathen V., Stapelmann K., von Woedtke T., Wende K. (2019). Nitrosylation vs. oxidation—How to modulate cold physical plasmas for biological applications. PLoS ONE.

[42] Bruno G., Wenske S., Lackmann J.W., Lalk M., von Woedtke T., Wende K. (2020). On the Liquid Chemistry of the Reactive Nitrogen Species Peroxynitrite and Nitrogen Dioxide Generated by Physical Plasmas. Biomolecules.

[43] Lukes P., Dolezalova E., Sisrova I., Clupek M. (2014). Aqueous-phase chemistry and bactericidal effects from an air discharge plasma in contact with water: Evidence for the formation of peroxynitrite through a pseudo-second-order post-discharge reaction of H_2_O_2_ and HNO_2_. Plasma Sources Sci. Technol..

[44] Jablonowski H., Schmidt-Bleker A., Weltmann K.D., von Woedtke T., Wende K. (2018). Non-touching plasma-liquid interaction—Where is aqueous nitric oxide generated?. Phys. Chem. Chem. Phys..

[45] Tresp H., Hammer M.U., Weltmann K.-D., Reuter S. (2013). Effects of Atmosphere Composition and Liquid Type on Plasma-Generated Reactive Species in Biologically Relevant Solutions. Plasma Med..

[46] Fiedler T.J., Davey C.A., Fenna R.E. (2000). X-ray crystal structure and characterization of halide-binding sites of human myeloperoxidase at 1.8 A resolution. J. Biol. Chem..

[47] Colas C., Ortiz de Montellano P.R. (2003). Autocatalytic radical reactions in physiological prosthetic heme modification. Chem. Rev..

[48] Karimi M., Crossett B., Cordwell S.J., Pattison D.I., Davies M.J. (2020). Characterization of disulfide (cystine) oxidation by HOCl in a model peptide: Evidence for oxygen addition, disulfide bond cleavage and adduct formation with thiols. Free Radic. Biol. Med..

[49] Kramer A.C., Torreggiani A., Davies M.J. (2017). Effect of Oxidation and Protein Unfolding on Cross-Linking of beta-Lactoglobulin and alpha-Lactalbumin. J. Agric. Food Chem..

[50] Grishkovskaya I., Paumann-Page M., Tscheliessnig R., Stampler J., Hofbauer S., Soudi M., Sevcnikar B., Oostenbrink C., Furtmuller P.G., Djinovic-Carugo K. (2017). Structure of human promyeloperoxidase (proMPO) and the role of the propeptide in processing and maturation. J. Biol. Chem..

[51] Bodnar Y., Lillig C.H. (2023). Cysteinyl and methionyl redox switches: Structural prerequisites and consequences. Redox Biol..

[52] Vakhrusheva T.V., Sokolov A.V., Kostevich V.A., Vasilyev V.B., Panasenko O.M. (2018). Enzymatic and Bactericidal Activity of Monomeric and Dimeric Forms of Myeloperoxidase. Biochem. Suppl. Ser. B Biomed. Chem..

[53] Heinecke J.W., Li W., Francis G.A., Goldstein J.A. (1993). Tyrosyl radical generated by myeloperoxidase catalyzes the oxidative cross-linking of proteins. J. Clin. Investig..

[54] Lund M.N., Luxford C., Skibsted L.H., Davies M.J. (2008). Oxidation of myosin by haem proteins generates myosin radicals and protein cross-links. Biochem. J..

[55] Andrekopoulos C., Zhang H., Joseph J., Kalivendi S., Kalyanaraman B. (2004). Bicarbonate enhances alpha-synuclein oligomerization and nitration: Intermediacy of carbonate radical anion and nitrogen dioxide radical. Biochem. J..

[56] Mukherjee S., Kapp E.A., Lothian A., Roberts A.M., Vasil’ev Y.V., Boughton B.A., Barnham K.J., Kok W.M., Hutton C.A., Masters C.L. (2017). Characterization and Identification of Dityrosine Cross-Linked Peptides Using Tandem Mass Spectrometry. Anal. Chem..

[57] Al-Hilaly Y.K., Williams T.L., Stewart-Parker M., Ford L., Skaria E., Cole M., Bucher W.G., Morris K.L., Sada A.A., Thorpe J.R. (2013). A central role for dityrosine crosslinking of Amyloid-beta in Alzheimer’s disease. Acta Neuropathol. Commun..

[58] Zeng L., Mathew A.V., Byun J., Atkins K.B., Brosius F.C., Pennathur S. (2018). Myeloperoxidase-derived oxidants damage artery wall proteins in an animal model of chronic kidney disease-accelerated atherosclerosis. J. Biol. Chem..

[59] Baldus S., Eiserich J.P., Brennan M.L., Jackson R.M., Alexander C.B., Freeman B.A. (2002). Spatial mapping of pulmonary and vascular nitrotyrosine reveals the pivotal role of myeloperoxidase as a catalyst for tyrosine nitration in inflammatory diseases. Free Radic. Biol. Med..

[60] Beckmann J.S., Ye Y.Z., Anderson P.G., Chen J., Accavitti M.A., Tarpey M.M., White C.R. (1994). Extensive nitration of protein tyrosines in human atherosclerosis detected by immunohistochemistry. Biol. Chem. Hoppe Seyler.

[61] Vlasak J., Bussat M.C., Wang S., Wagner-Rousset E., Schaefer M., Klinguer-Hamour C., Kirchmeier M., Corvaia N., Ionescu R., Beck A. (2009). Identification and characterization of asparagine deamidation in the light chain CDR1 of a humanized IgG1 antibody. Anal. Biochem..

[62] Yang H., Zubarev R.A. (2010). Mass spectrometric analysis of asparagine deamidation and aspartate isomerization in polypeptides. Electrophoresis.

[63] Doyle H.A., Gee R.J., Mamula M.J. (2007). Altered immunogenicity of isoaspartate containing proteins. Autoimmunity.

[64] Mamula M.J., Gee R.J., Elliott J.I., Sette A., Southwood S., Jones P.J., Blier P.R. (1999). Isoaspartyl post-translational modification triggers autoimmune responses to self-proteins. J. Biol. Chem..

