# Supplementary material for: Multi-Oxidant Environment as a Suicidal Inhibitor of Myeloperoxidase
Source: Antioxidants (Basel). 2023 Oct 30;12(11):1936. doi: 10.3390/antiox12111936 (PMC10668958; doi:10.3390/antiox12111936)
Supplement: Supplementary file 1 [file antioxidants-12-01936-s001.zip › MPO supplierí»s datasheet.pdf]

Calbiochem®

Certificate of Analysis

Myeloperoxidase, Human Polymorphonuclear Leukocytes

Batch Number: 3491204
Material Number: 475911-100UG
Molecular Weight: 150000
CAS Number: 9003-99-0
E.C. Number: 1.11.1.7

Quality Release Date: 07 AUG 2020
Recommended Retest Date: 31 JAN 2025

Analytical Data

Test	Tolerance	Results
**Formulation:**	Lyophilized in 50mM sodium acetate, pH 6.0, 100 – 700mM NaCl	Lyophilized in 50mM sodium acetate, pH 6.0, 100–700mM NaCl
**Solubility:**	H_2O (volume is lot specifics and can be found on the product label).<br><br>Further dilute in 100 mM NaCl, 50mM sodium acetate, pH 6.0<br><br>Enzyme may be precipitate out of solution in low ionic strength buffers or in distilled H_2O.	H_2O (volume is lot specifics and can be found on the product label).<br><br>Further dilute in 100mM NaCl, 50mM sodium acetate, pH 6.0<br><br>Enzyme may be precipitate out of solution in low ionic strength buffers or in distilled H_2O.
**Form:**	Solid	Solid
**"HBsAg; HIV; HCV antibodies":**	Negative	Negative
**Reconstitution:**	≥0.0 µL	17.1 µL
**Specific Activity:**	≥150.0 U/mgP	>200 U/mgP
**Purity by SDS-PAGE:**	≥95.0 %	95.0 %

Unit Definition: One unit is defined as the amount of enzyme that will reduce 1.0 µmol H_2O_2 per min at 25°C, pH 6.0.

Storage and Handling: -20°C

This lot conforms to specifications established by EMD Millipore Corporation for this product.

Quality Control/ Assurance Signature

07 AUG 2020
Date

Prices and availability are subject to change. ©Copyright 2020 EMD Millipore Corporation, an affiliate of Merck KGaA, Darmstadt, Germany. All rights reserved. Each product is sold with a limited warranty, which is provided with each purchase. Each product is intended to be used for research purposes only. It is not to be used for drug or diagnostic purposes, nor is it intended for human use. EMD Millipore products may not be resold, modified for resale, or used to manufacture commercial products without written approval of EMD Millipore.

EMD Millipore Corporation | 28820 Single Oak Dr., Temecula, CA 92590

Technical Support NA +1-800-221-1975 | email: www.millipore.com/techservices | www.calbiochem.com
Technical Support All Other Countries - Contact Your Local Office

FOR RESEARCH USE ONLY.

Not for use in diagnostic procedures. Not for human or animal consumption. Purchase of this product does not include any right to resell or transfer, either as a stand-alone product or as a component of another product. Any use of this Product for purpose other than research is strictly prohibited.

Calbiochem and all other trademarks, unless specifically identified as belonging to a third party, are owned by Merck KGaA, Darmstadt, Germany
