# Supplementary material for: Multi-Oxidant Environment as a Suicidal Inhibitor of Myeloperoxidase
Source: Antioxidants (Basel). 2023 Oct 30;12(11):1936. doi: 10.3390/antiox12111936 (PMC10668958; doi:10.3390/antiox12111936)
Supplement: Supplementary file 1 [file antioxidants-12-01936-s001.zip › Supplementary figures.pdf]

Supplementary Materials

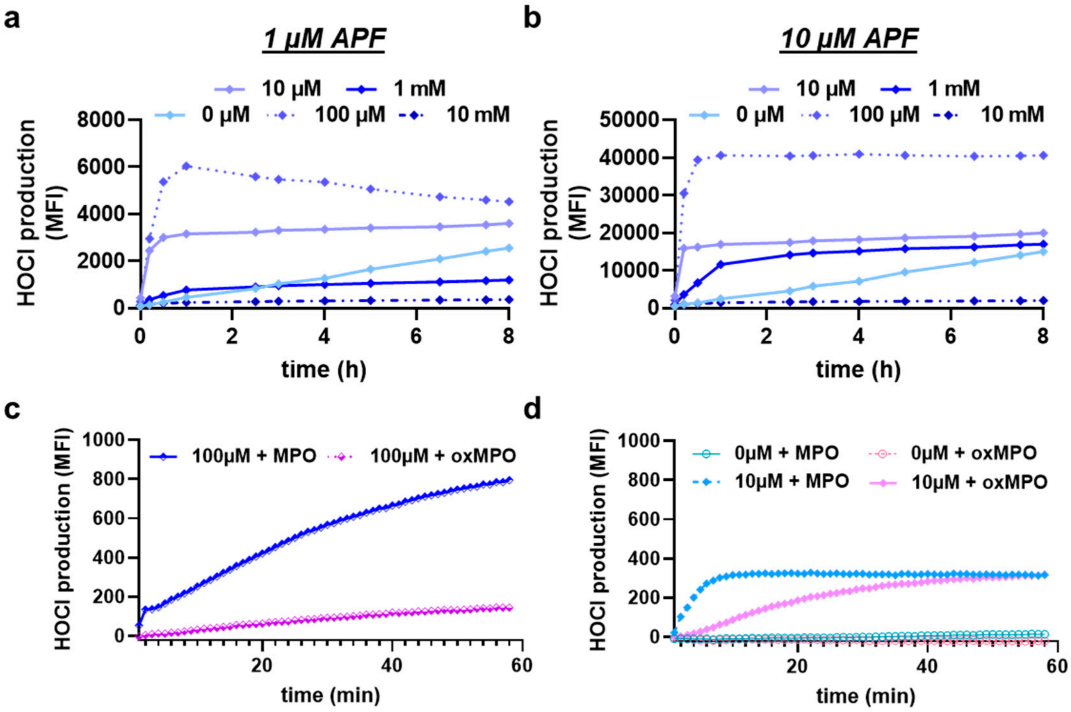

Figure S1. MPO activity is dependent on H_2O_2 and APF concentration. MPO was incubated with 0, 10, 100, 1000 or 10000 µM H_2O_2 to measure HOCl production in the presence of (**a**) 1 µM APF or (**b**) 10 µM APF. (**c, d**) MPO and plasma-treated MPO were incubated with different concentrations of H_2O_2 to measure HOCl production.

modification	mass shift (m/z)	amino acids	fine control
**+1O**	15.9949	A. D. F. G. H. I. L. P. S. T. V. Y. C. E. K. M. N. Q. W. R	common
**+2O**	31.9898	F. H. I. L. P. S. T. V. Y. C. E. K. M. N. Q. W. R	common
**+3O**	47.9847	F. H. I. L. P. Y. C. E. K. M. N. Q. W. R	common
**-2H+1O**	13.9792	A. D. H. I. L. P. S. T. V. Y. C. E. K. M. N. Q. W. R	common
**-1H-1N+1O**	0.98401	Q. N	common
**-1H+1N+2O**	44.9850	A. D. F. G. H. I. L. P. S. T. V. Y. C. E. K. M. N. Q. W. R	common
**-1H+1N+1O**	28.9901	C. K. M. N. P. Q. W. Y. F	common
**-1H+1N+3O**	60.9799	F. I. L. Y. W	common
**-1H+1N+4O**	76.9749	F. Y. W	common
**-2H+2O**	29.9741	F. H. I. L. P. R. S. V. Y. C. K. M. Q. W	common
**-2H+3O**	45.9690	F. I. K. W. Y	common
**-1C-1H-1N+2O**	4.97893	H	common
**-1H+1Cl**	33.9610	R. N. Q. H. K. F. W. Y	common
**-2H**	-2.01565	A. D. F. H. I. L. P. S. T. V. Y. C. E. K. M. N. Q. W. R	common
**-4H**	-4.03130	I. K. L. P. Q. S. T. V. W	common
**-1C-2H-1O**	-30.0105	D. E. P	common
**-1C-5H-3N+1O**	-43.0534	R	common
**-1S**	-31.9720	C	common
**-2H-1S**	-33.9877	C	common
**-1S+1O**	-15.9771	C	common
**-2C-1H-1N+1O**	-23.0159	H	common
**-2C-2H-2N+2O**	-22.0319	H	common
**-1C-2H-2N+2O**	-10.0319	H	common
**-3H-1N+1O**	-1.03163	K	common
**+1C+2O**	43.9898	K	common
**+1C+1H+1N+1O**	43.0058	K. C. D. E	common
**-1C-4H-1S+1O**	-32.0084	M	common
**-1C-2H+3O**	33.9690	M	common
**-1C+1O**	3.99491	W	common
**-1C+2O**	19.9898	W	common
**-4H+2O**	27.9585	W	common
**-4H+3O**	43.9534	W	common
**+4O**	63.9796	W	common
**-2H+2Cl**	67.9220	W. Y. F	common
**-2H+2N+4O**	89.9701	W. Y. F	common

Figure S2. List of modifications used for PMI-Byonic.

Ar		
**T mean**	**oxPTM**	**residue**
0.85	+1O	M577
0.73	+1O	M453
0.67	+1O	M509
0.53	+1O	M588
0.45	+1O	M341
0.38	+1O	M472
0.36	+1O	M631
0.15	+1O	F222
0.14	+1O	L539
0.14	+2O	C704
0.13	+2O	L539
1.00	+3O	C663
0.51	+3O	C704
0.42	+3O	C309
0.37	+3O	C606
0.10	-1C+1O	W535
0.15	-1C-4H-1S+1O	M577
0.26	-1H-1N+1O	N331
0.11	-2H+1O	M253
0.10	-2H+1O	Y723

Ar/O_2		
**T mean**	**oxPTM**	**residue**
0.90	+1O	M688
0.67	+1O	M453
0.54	+1O	M519
0.53	+1O	M551
0.50	+1O	M409
0.44	+1O	M341
0.40	+1O	M644
0.35	+1O	M415
0.32	+1O	M253
0.31	+1O	M588
0.19	+1O	L539
0.16	+1O	F222
0.13	+1O	P238
0.27	+2O	M577
0.25	+2O	M719
0.18	+2O	C704
0.61	+3O	C398
0.36	+3O	C704
0.15	-1C+1O	W535
0.19	-1C-4H-1S+1O	M588
0.15	-1H-1N+1O	N331
0.14	-2H	L539
0.15	-2H+1O	W535
0.48	-2H-1S	C309
0.48	-2H-1S	C606
0.13	-2H+1O	M341

ArN_2		
**T mean**	**oxPTM**	**residue**
0.42	+3O	C309
0.57	+3O	C398
0.37	+3O	C606
1.00	+3O	C663
0.14	+2O	C704
0.51	+3O	C704
0.15	+1O	F222
0.14	+1O	L539
0.13	+2O	L539
0.11	-2H+1O	M253
0.45	+1O	M341
0.73	+1O	M453
0.38	+1O	M472
0.67	+1O	M509
0.85	+1O	M577
0.15	-1C-4H-1S+1O	M577
0.53	+1O	M588
0.36	+1O	M631
0.26	-1H-1N+1O	N331
0.10	-1C+1O	W535
0.10	-2H+1O	Y723

Figure S3. Oxidative posttranslational modification found on MPO after plasma exposure. MPO was exposed to gas (control) or plasma, digested with trypsin and measured by Q-Exactive. The significance of the modification compared to gas control was determined using a custom R script that performed a Wilcoxon rank sum test with a cutoff of $p \leq 0.05$ (T mean).
